# Chinese herbal medicine for patients with atrial fibrillation: protocol for a systematic review and meta-analysis

**DOI:** 10.1097/MD.0000000000009228

**Published:** 2017-12-15

**Authors:** Xiaoli Cai, Yuanping Wang, Ziqing Li, Yu Zhang, Dawei Wang, Xia Yan

**Affiliations:** a Guangzhou University of Chinese Medicine; b The Second Affiliated Hospital of Guangzhou University of Chinese Medicine, Guangdong Provincial Hospital of Chinese Medicine; c Guangdong Provincial Key Laboratory of Research on Emergency in TCM, Guangzhou, China.

**Keywords:** atrial fibrillation, Chinese herbal medicine, protocol, systematic review

## Abstract

**Background:**

Atrial fibrillation (AF) is the most clinically common cardiac arrhythmia. Chinese herbal medicine (CHM) has been widely applied in the treatment of AF, However, to our knowledge, there has been no systematic review and meta-analysis of randomized controlled trails (RCTs) regarding the effectiveness of this treatment. Therefore, we provide a protocol to evaluate the effectiveness and safety of CHM for AF.

**Methods:**

The databases reviewed to collect RCTs related to CHM treatment for AF will be as follows: 3 English literature databases, which are PubMed, Embase, and Cochrane Library, and 3 Chinese literature databases, which are CBM, CNKI, and Wanfang. The data collection in the above-mentioned databases will be from the time when the respective databases were established to December 2017. The maintenance of sinus rhythm and p-wave dispersion will be accepted as the primary outcomes. Quality of life (QOL), such as QOL scale embolic events, bleeding events, and symptom improvement (such as chest distress, palpitations, etc) will be measured as secondary outcomes. Two reviewers will independently screen the titles, abstracts, or even full texts, and extract data. Methodological quality will be evaluated according to the Cochrane risk of bias. All analyses will be applied by RevMan (version 5.3).

**Results:**

The results of study will be disseminated via both international conference and peer-review journal.

**Conclusion:**

The conclusion of our systematic review will provide evidence to judge whether CHM is an effective intervention for patient with AF.

## Abstract 2

Supplemental Digital Content is available in the text

## 1 Introduction

Atrial fibrillation (AF) is the most clinically common cardiac arrhythmia, with 1% to 3% of the Europe general population suffering from the disease.^[1]^ Its prevalence varies among different continents and races, and the number of the patients with AF around the world is estimated to be 30 to 100 million.^[2]^ Studies have shown that the number will significantly raise in the next 30 to 50 years^[3,4]^ due to the aging of the population and increase in patients with arterial hypertension^[5]^ and diabetes,^[6]^ which both are the risk factors of AF. The existing studies have demonstrated that the prevalence and incidence of the disease among men are higher than that among women and increase with age.^[7]^ The AF is an important risk factor for the thromboembolic events, especially ischemic stroke. According to the epidemiological studies, patients with AF are 5 times as likely to have a stroke.^[8]^

AF can be divided into paroxysmal, persistent, and permanent AF based on the duration. The principle of AF treatment is to restore sinus rhythm, reduce the rapid ventricular rate, and prevent thrombosis and cerebral apoplexy.^[9]^ To date, antiarrhythmic drugs have still been the main treatment adopted for ventricular rate and rhythm control. The drugs are effective in the emergency treatment of AF, but the usage is limited by the recurrence of the disease and especially the inevitable side effects resulting from the long-term use, such as severe ventricular arrhythmias.^[10]^ Catheter ablation is an effective way to restore and maintain sinus rhythm in patients with AF.^[11,12]^ A systematic review showed that catheter ablation was more effective in maintaining sinus rhythm than antiarrhythmic drugs.^[13]^ However, the recurrence rate of the method is relatively high, and patients often need to undergo an operation again.^[14]^ Similarly, antithrombotic drugs also have their limitations, such as a high risk of bleeding, especially among elderly people.^[15]^

Chinese herbal medicine (CHM), which originated in ancient China and has long been based on traditional Chinese medicine (TCM), has been employed to treat AF in China for up to 2000 years.^[16]^ In modern China, traditional herbal formulas and Chinese patent medicines are used widely to treat patients with AF.^[17]^ A population-based cohort study with 70,698 patients as the subjects in Taiwan showed that about 1/8 of the patients with AF took TCM with the antithrombotic drugs at the same time.^[18]^ However, some studies also pointed out that most clinical trials on the clinical outcome and safety of TCM are limited by small sample size; hence, it is difficult to draw a reliable conclusion.^[19]^ In addition, it is not clear whether TCM can control ventricular rate, restore sinus rhythm, and prevent thromboembolic events in patients with AF. Although a meta-analysis indicated that, the curative effect of the combination of TCM and warfarin is better than that of warfarin alone in preventing the thromboembolic events among patients with AF, the conclusion should be treated with caution due to the average poor quality of the included studies.^[20]^

Given that there has not been any systematic review and meta-analysis report on the efficacy and safety of TCM treatment for AF, yet, it is necessary to deliver a systematic and comprehensive evaluation of the treatment to provide the clinicians, patients, and policy makers with a new therapy. This systematic review and meta-analysis will try to resolve the following four clinical questions about TCM treatment for AF: whether TCM is more effective than placebo; whether TCM is more effective than the conventional therapies, such as amiodarone and warfarin etc; (3) whether the TCM-assisted therapy is more effective than using TCM alone; and whether it is safe when TCM is used alone or combined with the conventional therapies.

## 2 Methods

### 2.1 Inclusion criteria for study selection

#### 2.1.1 Types of studies

All randomized controlled trials (RCTs) on TCM treatment for AF will be included in the study, without limit on language and publication status.

#### 2.1.2 Types of patients

All patients older than 18 and diagnosed with paroxysmal, persistent or permanent AF will be included in the study.^[21]^ There will be no restriction on sex or race, with animal studies excluded.

#### 2.1.3 Types of interventions

Interventions of the included trials will be oral administration of TCM, which can be a decoction, granules, or TCM with other modern dosage forms. The dosage and treatment course are not limited. The intervention of TCM with traditional western medicine can also be included. The control group will include placebo, blank control, and conventional medicine (such as an antiarrhythmic drug). If TCM is combined with western medicine (for instance, TCM is administrated with the antiarrhythmic drugs), the usage of western medicine in the control group should be consistent with that in the experimental group.

#### 2.1.4 Types of outcome measures

##### 2.1.4.1 Primary outcomes

- Maintenance of sinus rhythm
- P-wave dispersion

##### 2.1.4.2 Secondary outcomes

- Quality of life (QOL), such as QOL scale embolic events
- Embolic events
- Bleeding events
- Symptom improvement (such as chest distress, palpitations, etc)

#### 2.1.5 Search methods for the identification of studies

The databases reviewed to collect RCTs related to CHM treatment for AF will be as follows: 3 English literature databases, which are PubMed, Embase, and Cochrane Library, and 3 Chinese literature databases, which are CBM, CNKI, and Wanfang. The data collection in the above-mentioned databases will be from the time when the respective databases were established to December 2017. The retrieval strategy will be decided after several preretrievals in which the combination of medical keywords and uncontrolled terms is used. The searched terms will cover: AF, TCM, and random. At the same time, the references in the included trials and original literature included in the subject-related system evaluation will be attained as the supplementary literature to ensure the recall rate. Take PubMed as an example, its retrieval strategy will be shown in the Appendix 1.

#### 2.1.6 Searching other resources

In addition, the relevant conference papers will be manually retrieved. Moreover, the researchers will also contact the experts in the field and the corresponding author to obtain the important information that is not available through the above-mentioned data collection.

### 2.2 Data collection and analysis

#### 2.2.1 Selection of studies

The retrieved data will be imported into the literature management system of EndnoteX7 and the repetitive data will be removed by the corresponding researchers. They will also exclude the articles that are obviously not up to the standards by reading the titles and abstracts, and then determine the references that will be included eventually by reading through the text, discussing with the group members and contacting the author to learn about the details of the study. The final list of references will be translated into Microsoft Excel format. Two groups (2 researchers in each group) will each carry out the literature retrieval and literature screening independently. Lastly, the inconsistencies will be resolved by another study member, who will also check the final inclusion of literature.

#### 2.2.2 Data extraction and management

The software EpiData 3.1 will be employed to extract the data for double entry. Then another researcher will examine the data consistency and final database. Extracted data shall include: disease diagnosis, diagnosis/screening tools of AF, coexistent disease, course of disease, stage of disease, severity of disease, sample size, age, sex, intervention and the specific treatment in the control group, follow-up, outcome indicators, results, adverse events, and other details. The missing data, errors, and uncertainties will be resolved through group discussion, communication with the authors, or third-party arbitration.

#### 2.2.3 Assessment of risk of bias in included studies

The researchers will evaluate the risks from 7 aspects based on the Cochrane Collaboration's tool for assessing risk of bias in randomized trials provided by *Cochrane Handbook for Systematic Reviews of Interventions*, including random sequence generation, allocation concealment, blinding method for patients, researchers and outcomes assessors, incomplete result data, and selective reports. There will be low-risk, unclear, and high-risk evaluation results. Two trained researchers will independently conduct the evaluation. The inconsistencies will be resolved through group discussion, communication with the authors to confirm details, and arbitration with a third party.

#### 2.2.4 Measures of treatment effect

The enumeration data will be evaluated with the relative risk, whereas the measurement data the mean difference. A 95% confidence interval will be adopted to present the effect sizes.

#### 2.2.5 Dealing with missing data

The researchers will try to gain the missing or unavailable data via the corresponding author of the original articles. If that fails, the analysis will be conducted based on the available data.

#### 2.2.6 Assessment of heterogeneity

χ^2^ test (α = 0.1) and *I*^2^ value will be employed respectively to analyze and determine the heterogeneity of the research results. The statistic heterogeneity among trials can be considered negligible if *I*^2^ is ≤50%, and the fixed effects model will be used to calculate the effect size. On the contrary, the heterogeneity among the trials will be considered significant if *I*^2^ is >50%.

#### 2.2.7 Assessment of reporting bias

First, visual symmetry on a funnel plot will be generated to judge preliminarily whether a publication bias exists when >10 studies are included in the research. The quantitative analysis of the Egger test will be carried out using the software STATA 12.0 if the generated image is blurry.

#### 2.2.8 Data synthesis

The meta-analysis will be done with the software RevMan 5.3. If no statistic heterogeneity among the results of the study is found, the meta-analysis will be performed using the fixed effects model. However, the source of the heterogeneity should be further analyzed if a statistic heterogeneity is found. With the effect of the obvious clinical heterogeneity excluded, the meta-analysis will be conducted with the random effect model. The researcher can choose to carry out the subgroup or sensitivity analysis, or only descriptive analysis if there is obvious clinical heterogeneity.

#### 2.2.9 Subgroup analysis

The subgroup analysis will be performed based on different kinds of TCM and AF, the patient's age, sex, and treatment period if a significant heterogeneity is discovered in the included trials.

#### 2.2.10 Sensitivity analysis

**Figure 1:**
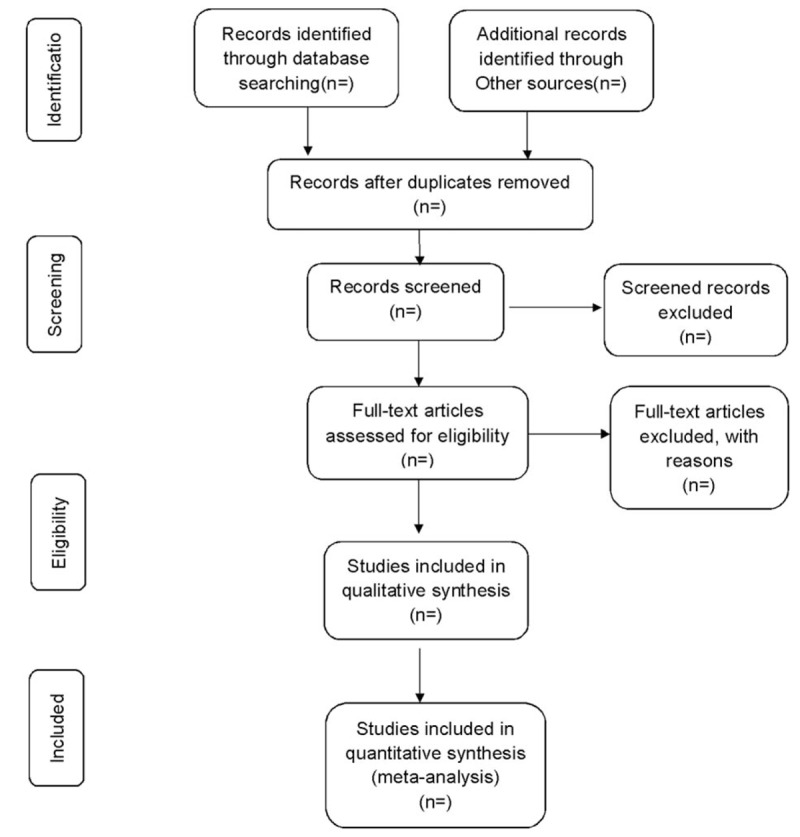
Preferred Reporting Items for Systematic review and Meta-Analysis (PRISMA) flow chart.

If enough trials are included, the researchers will conduct a sensitivity analysis of the main outcome indicators to examine the stability of the results. With the low-quality literature and studies of small sample size ruled out, the meta-analysis will be reconducted to assess whether these factors affect the results (Fig. 1).

#### 2.2.11 Grading the quality of evidence

It is recommended that the quality evaluation of the main results be carried out with Grading of Recommendations, Development and Evaluation. The results will be divided into four grades: high, medium, low, or very low.^[22]^

## 3 Discussion

Studies have shown that TCM is effective in improving the clinical symptoms of AF, such as chest congestion and palpitation, etc, and has relatively few side effects, but the exact mechanism is still to be further explored.^[23,24]^ However, there has been no English meta-analysis of TCM treatment for AF yet. The research will consist of 4 parts: identification of studies, selection of studies, data analysis, and data extraction and management. The researchers hope that this study will provide more convincing evidence to prove the advantages of TCM in treating AF. Nevertheless, there may be some potential shortcomings in this study. For example, the interventions of the included studies adopt different types of TCM, dosage, and course of treatment, which may cause great heterogeneity of the results of meta-analysis. In addition, it may lead to a certain bias as only studies published in English and Chinese will be searched and reviewed in the research.

Preferred Reporting Items for Systematic review and Meta-Analysis Protocols checklist of this protocol is presented in online supplementary.

## Supplementary Material

Supplemental Digital Content
