# Supplementary material for: Chinese herbal medicine for patients with atrial fibrillation: protocol for a systematic review and meta-analysis
Source: Medicine (Baltimore). 2017 Dec 15;96(50):e9228. doi: 10.1097/MD.0000000000009228 (PMC5815763; doi:10.1097/MD.0000000000009228)
Supplement: Supplemental Digital Content [file medi-96-e9228-s001.docx]

**Appendix 1. Search strategy used in PubMed database**

#1Atrial Fibrillations OR Fibrillation, Atrial OR Fibrillations, Atrial OR Auricular Fibrillation OR Auricular Fibrillations OR Fibrillation, Auricular OR Fibrillations, Auricular OR Persistent Atrial Fibrillation OR Atrial Fibrillation, Persistent OR Atrial Fibrillations, Persistent OR Fibrillation, Persistent Atrial OR Fibrillations, Persistent Atrial OR Persistent Atrial Fibrillations OR Familial Atrial Fibrillation OR Atrial Fibrillation, Familial OR Atrial Fibrillations, Familial OR Familial Atrial Fibrillations OR Fibrillation, Familial Atrial OR Fibrillations, Familial Atrial OR Paroxysmal Atrial Fibrillation OR Atrial Fibrillation, Paroxysmal OR Atrial Fibrillations, Paroxysmal OR Fibrillation, Paroxysmal Atrial OR Fibrillations, Paroxysmal Atrial OR Paroxysmal Atrial Fibrillations

#2 Traditional Chinese Medicine OR Chinese Traditional Medicine OR Chinese Herbal Drugs OR Chinese Drugs, Plant ORMedicine, Traditional OR Ethnopharmacology OR Ethnomedicine

OR Ethnobotany OR Medicine, Kampo OR Kanpo OR TCM ORMedicine, Ayurvedic OR Phytotherapy OR Herbology OR Plants,Medicinal OR Plant Preparation OR Plant Extract OR Plants, MedicineOR Materia Medica OR Single Prescription OR Herbs ORChinese Medicine Herb OR Herbal Medicine

#3 Randomized controlled trial OR clinical study OR Clin-ical Trial OR Controlled study OR Controlled Trial OR Random*Control* study OR random* Control* Trial

#1 AND #2 AND #3
